# Chemical Elicitors-Induced Variation in Cellular Biomass, Biosynthesis of Secondary Cell Products, and Antioxidant System in Callus Cultures of *Fagonia indica*

**DOI:** 10.3390/molecules26216340

**Published:** 2021-10-20

**Authors:** Habiba Khan, Tariq Khan, Nisar Ahmad, Gouhar Zaman, Taimoor Khan, Waqar Ahmad, Sannia Batool, Zahid Hussain, Samantha Drouet, Christophe Hano, Bilal Haider Abbasi

**Affiliations:** 1 Department of Biotechnology, Quaid-i-Azam University, Islamabad 45320, Pakistan; habibabiotech2019@gmail.com (H.K.); tariqkhanbio@gmail.com (T.K.); gouharkhan4400@gmail.com (G.Z.); taimoor@unizwa.edu.om (T.K.); waqar@unizwa.edu.om (W.A.); sanniabatool96@gmail.com (S.B.); 2 Department of Biotechnology, University of Malakand, Malakand 23050, Pakistan; 3 Center for Biotechnology and Microbiology (CB&M), University of Swat, Swat 19200, Pakistan; ahmadn@uswat.edu.pk (N.A.); zahid@uswat.edu.pk (Z.H.); 4 Natural and Medical Sciences Research Center, University of Nizwa, Nizwa 616, Oman; 5 Laboratoire de Biologie des Ligneux et des Grandes Cultures (LBLGC), INRA USC1328, Université d’Orléans, CEDEX 2, 45067 Orléans, France; samantha.drouet@univ-orleans.fr

**Keywords:** *Fagonia indica*, callus culture, chemical elicitors, polyphenolics, antioxidant enzymes, HPLC

## Abstract

*Fagonia indica* is a rich source of pharmacologically active compounds. The variation in the metabolites of interest is one of the major issues in wild plants due to different environmental factors. The addition of chemical elicitors is one of the effective strategies to trigger the biosynthetic pathways for the release of a higher quantity of bioactive compounds. Therefore, this study was designed to investigate the effects of chemical elicitors, aluminum chloride (AlCl_3_) and cadmium chloride (CdCl_2_), on the biosynthesis of secondary metabolites, biomass, and the antioxidant system in callus cultures of *F. indica*. Among various treatments applied, AlCl_3_ (0.1 mM concentration) improved the highest in biomass accumulation (fresh weight (FW): 404.72 g/L) as compared to the control (FW: 269.85 g/L). The exposure of cultures to AlCl_3_ (0.01 mM) enhanced the accumulation of secondary metabolites, and the total phenolic contents (TPCs: 7.74 mg/g DW) and total flavonoid contents (TFCs: 1.07 mg/g DW) were higher than those of cultures exposed to CdCl_2_ (0.01 mM) with content levels (TPC: 5.60 and TFC: 0.97 mg/g) as compared to the control (TPC: 4.16 and TFC: 0.42 mg/g DW). Likewise, AlCl_3_ and CdCl_2_ also promoted the free radical scavenging activity (FRSA; 89.4% and 90%, respectively) at a concentration of 0.01 mM, as compared to the control (65.48%). For instance, the quantification of metabolites via high-performance liquid chromatography (HPLC) revealed an optimum production of myricetin (1.20 mg/g), apigenin (0.83 mg/g), isorhamnetin (0.70 mg/g), and kaempferol (0.64 mg/g). Cultures grown in the presence of AlCl_3_ triggered higher quantities of secondary metabolites than those grown in the presence of CdCl_2_ (0.79, 0.74, 0.57, and 0.67 mg/g). Moreover, AlCl_3_ at 0.1 mM enhanced the biosynthesis of superoxide dismutase (SOD: 0.08 nM/min/mg-FW) and peroxidase enzymes (POD: 2.37 nM/min/mg-FW), while CdCl_2_ resulted in an SOD activity up to 0.06 nM/min/mg-FW and POD: 2.72 nM/min/mg-FW. From these results, it is clear that AlCl_3_ is a better elicitor in terms of a higher and uniform productivity of biomass, secondary cell products, and antioxidant enzymes compared to CdCl_2_ and the control. It is possible to scale the current strategy to a bioreactor for a higher productivity of metabolites of interest for various pharmaceutical industries.

## 1. Introduction

Plant-based products have piqued the interest of many nutraceutical and pharmaceutical companies, who prefer them to synthetic medications [1]. *Fagonia indica* is one of the most important medicinal plants that belong to the family *Zygophyllaceae* with restricted global distribution and can be found in several parts of the world, including Pakistan, India, and Afghanistan [2]. *F. indica* has been investigated extensively, and its therapeutic properties are well documented. Saponins, alkaloids, terpenoids, sterols, flavonoids, and trace elements are among the bioactive chemicals that give this plant its therapeutic effects [3]. Its multiple therapeutic properties include anti-inflammatory [4], hepato-protective [5], anticancer [6], anti-diabetic [7], antimicrobial [8], antioxidant [9], antihemorrhagic, anthelminthic, and thrombolytic features [10].

Due to the medical value of *F. indica*, there has been a significant increase in market demand for the plant in recent years. The natural supply of this valuable herb is insufficient to meet the rising demand. Alternative approaches for balancing the supply and demand chain should be pursued [11]. Plant cell factories have the advantage of establishing plant in vitro cultures for the production of continuous, consistent, and healthy plant material with long-term metabolite profiles [12]. The plant has been studied in vitro to generate essential phytochemicals using various culture systems, to increase the production of medicinally relevant secondary metabolites [11,13,14]. Elicitation in plant cell cultures has proven to be one of the most effective ways for increasing medicinal chemical production, and it has commercial implications [15,16]. In plant cell cultures, elicitors tend to trigger multiple physiological events that eventually lead to the activation of a cascade of reactions that include the expression of defense-related genes, the production of reactive oxygen species (ROS), and the accumulation of important secondary metabolites such as polyphenolics [17,18]. There are many different types of elicitors divided into two categories based on their nature or origin: biotic and abiotic elicitors [19].

Previously, elicitors were used to optimize the synthesis of medicinally important phytochemicals in numerous in vitro cultures, including callus and adventitious root cultures of *F. indica* [19,20,21]. In vitro cultures of *F. indica* have been used to activate the production of health-promoting secondary metabolites by adding elicitors or plant growth regulators such as methyl jasmonate (Me-J), polyacrylic acid (PAA), and melatonin [11,20]. In addition, in callus cultures, changes in carbohydrate supply and fungal-derived chitosan elicitors have been successfully used to elicit secondary metabolites in *F. indica* [19,20]. In addition to biotic elicitors, abiotic elicitors such as light, temperature, air, ultraviolet radiation (UV), pH variations, and heavy metal salts have been used to generate optimal metabolite concentrations in plant cell cultures over the last few decades [22,23].

Heavy metals among abiotic elicitors have been extensively utilized in several plant species to boost growth, phytochemicals accumulation, and antioxidant potential [24,25,26,27]. Previously, the salts of cadmium (Cd^2+^) (generally applied as cadmium chloride (CdCl_2_)) and aluminum chloride (AlCl_3_) have been reported for the improved production of compounds in the callus culture of *Rauvolfia serpentina* [28], suspension culture of *Melissa officinalis* L. [29], and cell culture of *Vitis vinifera* [30]. However, heavy metals are seen to possess toxicity in their application to plant cells under controlled conditions. Furthermore, plants have a powerful antioxidant defense system that produce higher quantities of phenolic compounds that, in turn, chelate metal ions and thus help in their sequestration [31].

AlCl_3_ has been shown to promote growth and stimulate secondary metabolites in plant cultures, as well as cope with reactive oxygen species (ROS) by promoting the production of antioxidative enzyme genes such as glutathione S-transferase, peroxidase (POD), and superoxide dismutase (SOD) [32,33,34].

However, no previous studies on the impact of heavy metal salts on the generation of medicinal substances in *F. indica* callus culture have been published. As a result, the current study was designed to show how AlCl_3_ and CdCl_2_ affect cellular biomass, secondary cell products, and the antioxidant system in *F. indica* callus cultures. In addition, the treated cultures were measured using high-performance liquid chromatography to determine the bioactive metabolites.

## 2. Results and Discussion

### 2.1. Abiotic Elicitors-Induced Variation in Cellular Biomass in Calli Cultures of F. indica

**Figure 1:**
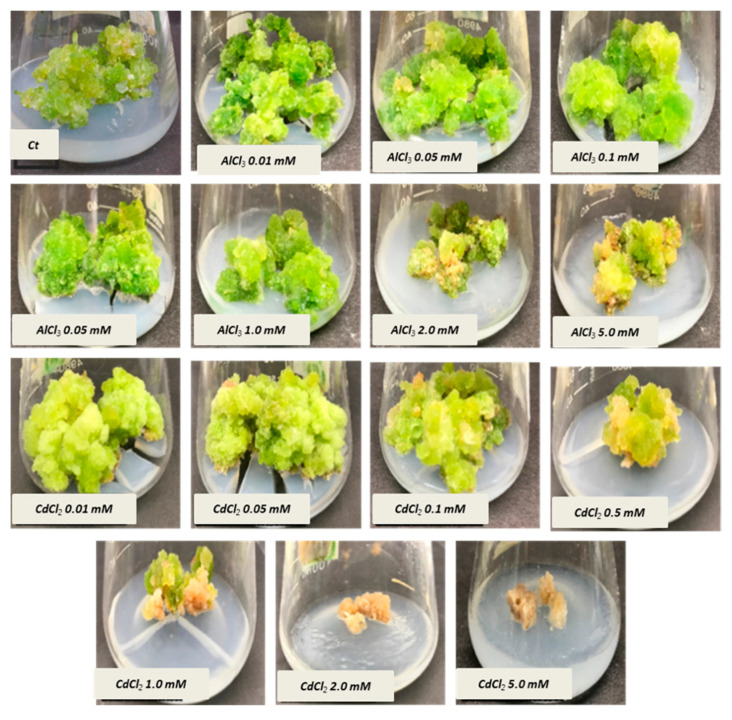
Morphological characteristics of calli incubated for 35 days under different concentrations of aluminum chloride (AlCl_3_) and cadmium chloride CdCl_2_ (color, texture, and morphology).

**Figure 2:**
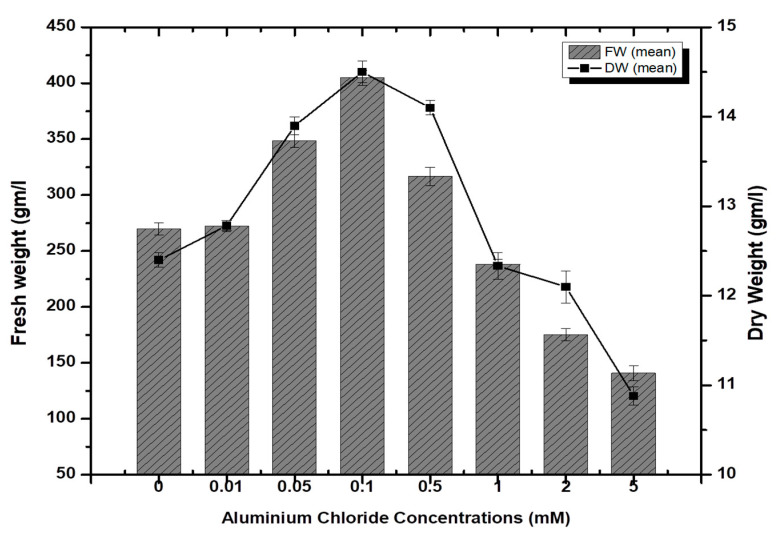
Aluminum chloride-elicited biomass accumulation (fresh and dry weight) in callus culture of *F. indica* under seven different concentrations along with control. All the values are mean ± SE.

**Figure 3:**
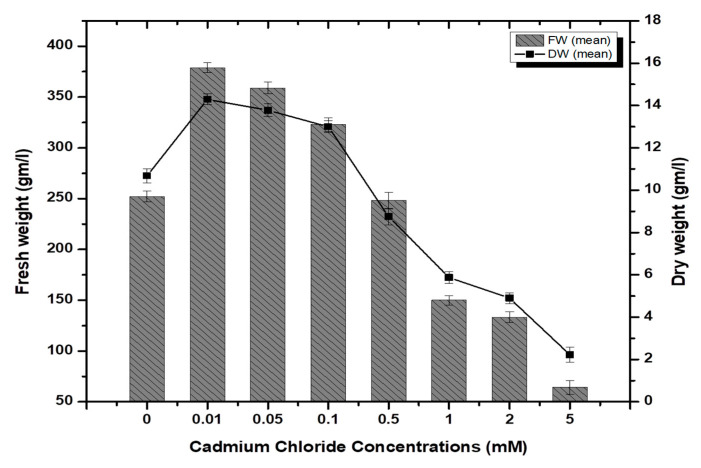
Cadmium chloride-elicited biomass accumulation (fresh and dry weight) in callus culture of *F. indica* under seven different concentrations along with control. All the values are mean ± SE.

Stem-derived callus cultures of *F. indica* optimized previously by our research group were analyzed for AlCl_3_ and CdCl_2_ salts’ effects on biomass accumulation. Our investigations showed that lower concentrations of both elicitors had growth-promoting effects (Figure 1). The maximum biomass fresh weight (FW: 404.7 g/L) and dry weight (DW: 14.5 g/l) was recorded in cultures inoculated on MS media augmented with AlCl_3_ (0.1 mM) as compared to the control (269.85 g/L). The biomass decreased with the concentration of AlCl_3_ (Figure 2). It is interesting to note that our results are in agreement with the previous reports [28,35], where lower concentrations of these elicitors are superior in inducing higher cellular biomass in plant cell cultures. Similarly, the maximum biomass accumulation induced by CdCl_2_ (0.01 mM) in terms of fresh weight (FW: 378.9 g/L) and dry weight (DW: 14.3 g/L) was comparatively lowered than AlCl_3,_ and likely, the minimum cellular biomass accumulation (64.3 g/L-FW and 2.21 g/L-DW) was recorded when cultures were exposed to 5.0 mM of CdCl_2_ (Figure 3). Furthermore, the callus cultures were also investigated for morphological features under AlCl_3_ and CdCl_2_ inoculations.

The callus cultures grown on both elicitors were observed to be compact and green at lower concentrations while friable and brown at the highest concentrations (Figure 1). This could be possibly due to the hypersensitivity induced by higher concentrations, i.e., leading to cell death [36,37]. In previous investigations, lower concentrations of CdCl_2_ resulted in increased biomass accumulation. In contrast, higher concentrations decreased several growth indices in plant cultures such as *Vigna radiata,* i.e., mung bean [38], *Vitis vinifera* cv. Cell suspension cultures [39], sugar cane callus cultures [40], hairy root cultures of *Brugmansia candida* [41], and roots of *Atropa belladonna* [23], and a marked decline in cell viability in *Nicotiana tabacum* L cells [42]. Many investigators have demonstrated inhibitory effects of heavy metals on plant growth by measuring various growth parameters such as root elongation, protein concentration, phenolic biosynthesis, and fresh and dry cellular biomass [43,44,45].

### 2.2. Effect of Elicitation on Total Phenolic and Flavonoid Biosynthesis

**Figure 4:**
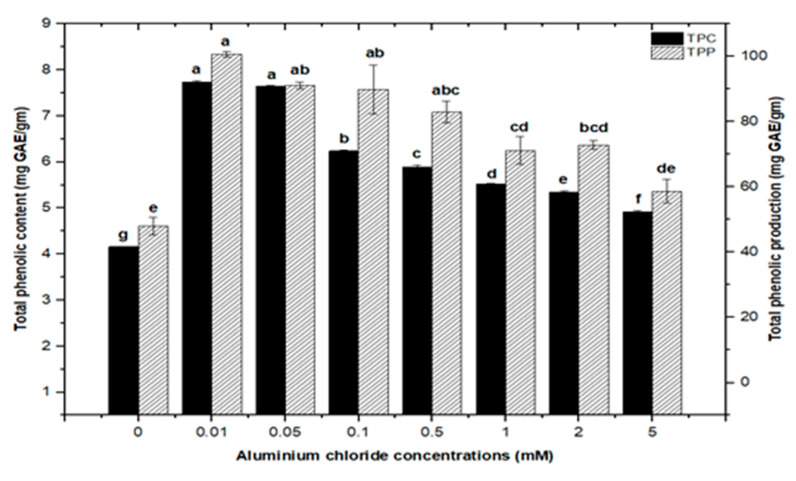
Total phenolic contents and phenolic production of *F. indica* calli elicited with seven different concentrations of aluminum chloride and control. All the values are mean ± SE. Bars labeled with different letters (LSD values) exhibit significant variation (α < 0.05).

**Figure 5:**
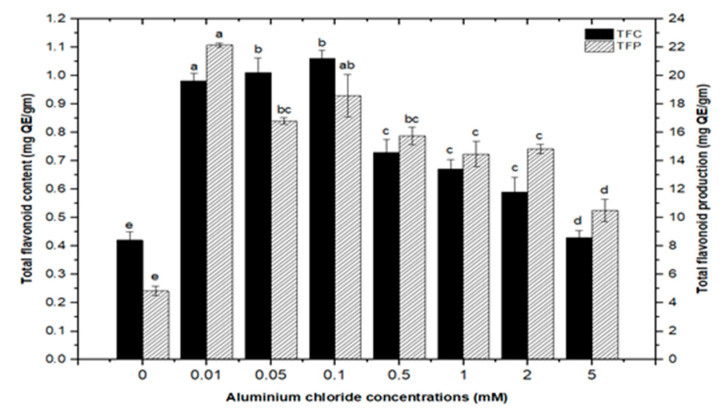
Total flavonoids contents and flavonoids production of *F. indica* calli elicited with seven different concentrations of aluminum chloride and control. All the values are mean ± SE. Bars labeled with different letters (LSD values) exhibit significant variation (α < 0.05).

High doses of elicitor have been shown to cause hypersensitivity and cell death, whereas a moderate level was required for optimal secondary metabolite induction [36,37]. Inorganic salts/chemicals such as AgNO^3^, CdCl_2_, AlCl_3_, and HgCl_2_ have been employed widely in various plant species to trigger and increase the production of bioactive molecules by altering secondary metabolism [27]. Callus cultures of *F. indica* elicited with different concentrations of AlCl_3_ and CdCl_2_ showed a varying effect on total phenolics accumulation. Cultures added with AlCl_3_ showed a maximum elicitation of total phenolic content (TPC) (7.74 mg/g DW) and (7.64 mg/g DW) at 0.01 and 0.05 mM of AlCl_3_, respectively, as compared with the control (4.159 mg/g DW) (Figure 4). Maximum levels for total flavonoid content (TFC) (1.069 mg/g DW), (1.014 mg/g DW), and (0.998 mg/g DW) were recorded at 0.1, 0.05, and 0.01 mM of AlCl_3_, respectively, while a further increase in AlCl_3_ resulted in a decrease in flavonoid productions as compared with the control (0.4225 mg/g DW) (Figure 5). Furthermore, for cultures elicited with AlCl_3_, maximum values for total flavonoid production (22.11 mg/L) and (18.6 mg/L) were observed at 0.01 and 0.1 mM of AlCl_3_, respectively (Figure 5). In a similar study on root cultures of *Gloriosa superba*, AlCl_3_ greatly enhanced the phenolic and flavonoid content, as well as the production of colchicine [35]. An increase in reserpine content in response to low doses of AlCl_3_ has also been reported [28].

**Figure 6:**
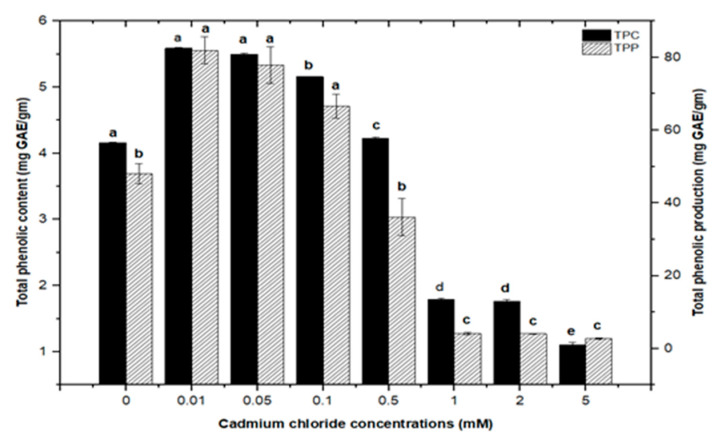
Total phenolic contents and phenolic production of *F. indica* calli elicited with seven different concentrations of cadmium chloride and control. All the values are mean ± SE. Bars labeled with different letters (LSD values) exhibit significant variation (α < 0.05).

Similarly, callus cultures subjected to CdCl_2_ treatment had maximum total phenolic contents of (5.590 mg/g DW) and (5.501 mg/g DW) at 0.01 and 0.05 mM, respectively, with a similar maximum value (5.156 mg/g DW) at 0.1 mM of CdCl2. CdCl_2_ demonstrated a substantial inhibitory impact at higher concentrations, with the lowest total phenolic content (1.104 mg/g DW) detected at 5.0 mM, compared to total phenolic content (1.792 mg/g DW) and (1.762 mg/g DW) at 1.0 and 2.0 mM, respectively. The total phenolic synthesis in callus cultures was triggered with CdCl_2_ doses, with the highest value (81.911 mg/L) seen at 0.01 mM of CdCl_2_, followed by a similar maximum value (77.94 mg/L) at 0.05 mM of CdCl_2_ (Figure 6).

**Figure 7:**
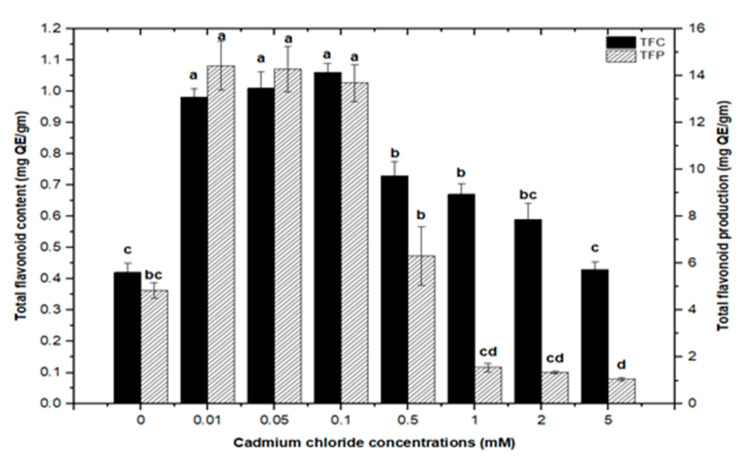
Total flavonoids contents and flavonoids production of *F. indica* calli elicited with seven different concentrations of cadmium chloride and control. All the values are mean ± SE. Bars labeled with different letters (LSD values) exhibit significant variation (α < 0.05).

Furthermore, adding greater concentrations of CdCl_2_ to callus cultures (0.5, 0.1, 2.0, and 5 mM) caused evident toxicity and a decrease in total phenolic synthesis. Maximum comparable values for total flavonoid contents (1.062 mg/g DW), (1.010 mg/g DW), and (0.976 mg/g DW) were found at respective treatments of 0.1, 0.05, and 0.01 mM of CdCl_2_, as compared with the control. The higher concentrations of CdCl_2_ (0.5, 1.0, and 2.0 mM of CdCl_2_) resulted in maximum possible flavonoids accumulations (0.730 mg/g DW), (0.672 mg/g DW), and (0.591 mg/g DW), respectively. However, CdCl_2_ at 5.0 mM resulted in the least value for total flavonoid content (0.432 mg/g DW), closely similar to that of the control with (0.4225 mg/g DW) of total flavonoid content (Figure 7). For total flavonoid production in CdCl_2_-treated calli, maximum values for total flavonoid production (14.322 mg/L), (14.306 mg/L), and (13.715 mg/L) were observed at 0.01, 0.05, and 0.1 mM of CdCl_2_. Previously, many studies have shown CdCl_2_ as an effective elicitor for optimum phytochemicals production in in vitro cultures of *Salvia miltiorrhiza* [18], *Catharanthus roseus* [46], and *Datura stramonium* [47]. Similar results were also reported by [30,39].

### 2.3. Correlation of Total Phenolics and Flavonoids Content with Radical Scavenging Activity

**Figure 8:**
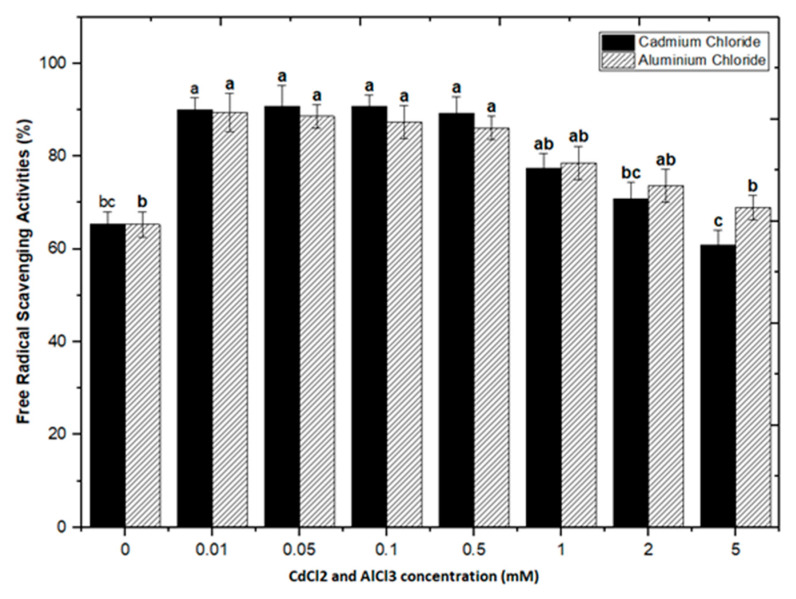
DPPH free radicals scavenging activity of *F. indica* calli elicited with seven different concentrations of cadmium chloride and aluminum chloride and control. All the values are mean ± SE. Bars labeled with different letters (LSD values) exhibit significant variation (α < 0.05).

Natural antioxidants are vital substances that can protect organisms from damage caused by oxidative stress generated by free radicals [48]. As a result, plants have evolved a variety of defense measures (antioxidant system) to scavenge the harmful radicals created during oxidative stress, allowing them to survive [13,28,30,49,50]. Variable antioxidant activity in response to heavy metals (AlCl_3_ and CdCl_2_) was examined to understand better the influence of heavy metal elicitors on antioxidant activity. Calli elicited with AlCl_3_ and CdCl_2_ significantly enhanced the radical scavenging activity by approximately 30% with respect to control cultures (Figure 8). The addition of lower concentrations of AlCl_3_ to the incubation medium caused a significant increase in antioxidant activity. It showed maximum activities of 89.40%, 88.60%, 87.40%, and 86.10% at 0.01, 0.05, 0.1, and 0.5 mM of AlCl_3_, respectively, as compared to the control (65.30%).

**Figure 9:**
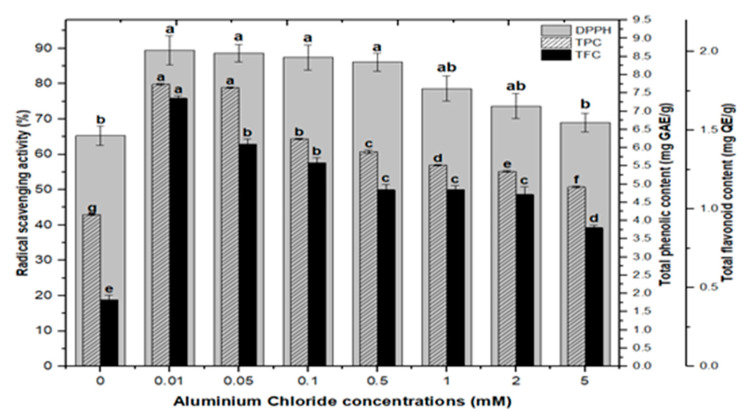
Radical scavenging activity (DPPH %) in comparison with total phenolic contents (TPCs) and total flavonoid contents (TFCs) (correlation ‘r’ DPPH vs. TPC = 0.815 and DPPH vs. TFC = 0.838) in *F. indica* callus culture elicited with seven different concentrations of aluminum chloride along with control. Bars labeled with different letters (LSD values) exhibit significant variation (α < 0.05).

**Figure 10:**
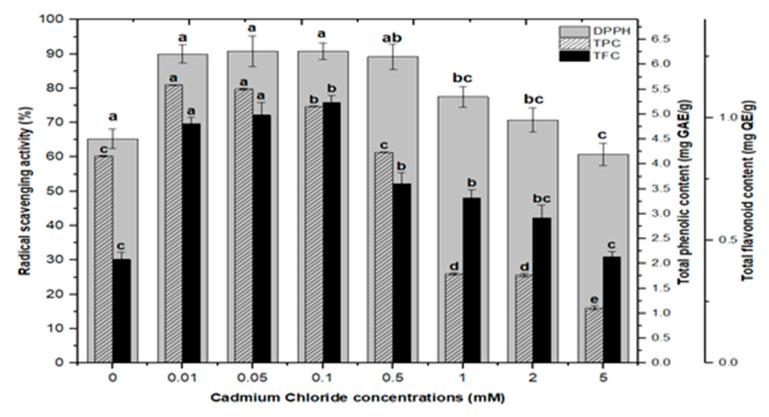
Radical scavenging activity (DPPH %) in comparison with total phenolic contents (TPCs) and total flavonoid contents (TFCs) (correlation ‘r’ DPPH vs. TPC = 0.727 and DPPH vs. TFC = 0.925) in *F. indica* callus cultures elicited with seven different concentrations of cadmium chloride along with control. Bars labeled with different letters (LSD values) exhibit significant variation (α < 0.05).

Lower doses of CdCl_2_ resulted in maximum activities of 90.0%, 90.8%, 90.8% and 89.2% in cultures, respectively, at 0.01, 0.05, 0.1, and 0.5 mM of CdCl_2_. Higher AlCl_3_ and CdCl_2_ concentrations (1, 2, and 5 mM) increased the antioxidant activity less or not at all compared to the control (Figure 8). The radical scavenging activity of both AlCl_3_ and CdCl_2_ was found to be strongly correlated with the increase in phenolic and flavonoid contents (Figure 9 and Figure 10). The plants either use antioxidant enzymes to scavenge the toxic products of ROS or the synthesis of compounds to combat stress conditions. Currently, the addition of AlCl_3_ and CdCl_2_ induced stress conditions. Therefore, the plant cell activated the defense system and released polyphenolics, especially phenolics and flavonoids that protect the plant cells from damaging agents. Thus, these salts are directly correlated with the defense system of plants and activate it to produce phenolics to protect plant cells.

### 2.4. Effect of AlCl_3_ and CdCl_2_ on Antioxidant Enzyme Activities

Cellular membranes, nucleic acids, proteins, lipids, and chlorophyll can be damaged by reactive oxygen species (ROS). As four principal active oxygen species formed in plant tissues, the most common ROSs are O_2_ (superoxide radical), H_2_O_2_ (hydrogen peroxide), OH (hydroxyl radical), and singlet oxygen [30,51,52]. Plants have evolved a well-organized anti-oxidative enzymatic system to deal with stress and damage caused by ROS, with superoxide dismutase (SOD) and peroxidase (POD) serving as the first line of defense [43,45,53]. In this study, the activities of SOD and POD were measured in callus cultures of *F. indica* to assess their role in heavy metal (AlCl_3_ and CdCl_2_) salt stress. Our research revealed that callus cultures had stronger superoxide dismutase (SOD) and peroxidase (POD) activities than control cultures did.

**Figure 11:**
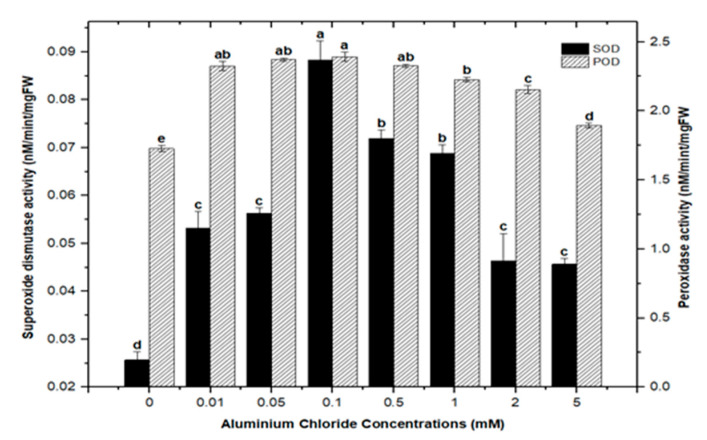
Superoxide dismutase and peroxidase activity of *F. indica* calli elicited with seven different concentrations of aluminum chloride along with the control. Bars labeled with different letters (LSD values) exhibit significant variation (α < 0.05).

**Figure 12:**
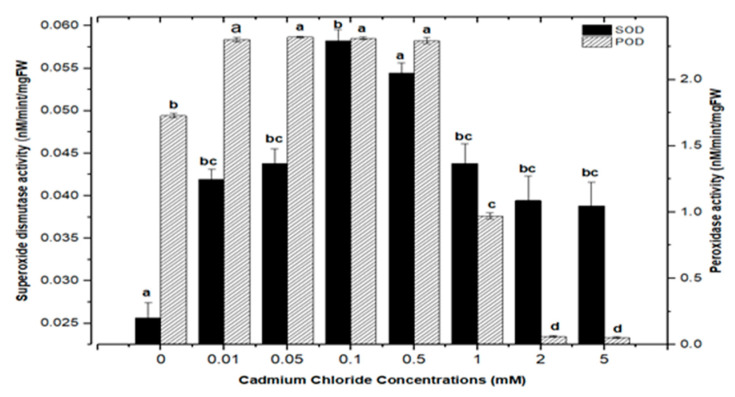
Superoxide dismutase and peroxidase activity of *F. indica* calli elicited with seven different concentrations of cadmium chloride along with control. Bars labeled with different letters (LSD values) exhibit significant variation (α < 0.05).

Highest superoxide dismutase (SOD: 0.088 nM/min/mg FW) and peroxidase enzyme activities (POD: 2.372 nM/min/mg FW) were recorded in culture with AlCl_3_ elicitation (concentrations of 0.1 mM), respectively, in comparison with the control: SOD: 0.025 and POD: 1.69 nM/min/mg FW (Figure 11). Similarly, the antioxidant enzyme activities for cultures inoculated with CdCl_2_ were SOD (0.058 nM/min/mg FW) and POD (2.721 nM/min/mg FW) with CdCl_2_ elicitation at a 0.1 mM concentration and CdCl_2_ (0.5 mM) respectively, when compared to the control (Figure 12). The increased enzymatic activities in cultures provoked with heavy metal salts have been previously proved to be due to inducing ROSs that further increase the expression levels of several genes encoding antioxidative enzymes such as glutathione S-transferase and peroxidase, and superoxide dismutase [45,54,55].

Moreover, the least activities observed were (SOD: 0.045 nM/min/mg FW and POD: 1.895 nM/min/mg FW) in AlCl_3_ and (SOD: 0.038 nM/min/mg FW and POD: 0.053 nM/min/mg FW) with CdCl_2_ recorded at a concentration of (5.0 mM) of both elicitors (Figure 11 and Figure 12). Overall, lower doses of both metals as elicitors resulted in increased activities, whereas higher concentrations inhibited the lowest SOD and POD activities. Inactivation of the enzymes due to overproduction of ROS or inactivation of the enzyme by H_2_O_2_ in various compartments could explain the reduction in enzymes at higher metal concentrations [56]. Similar results have been reported in different plants where a specific level of Cd produces increases in SOD and POD activities, with higher increases causing a drop in enzymatic activities [52,55,57,58,59,60].

### 2.5. Quantification of the Main Phytochemicalsin-Treated Callus Cultures of F. indica

**Figure 13:**
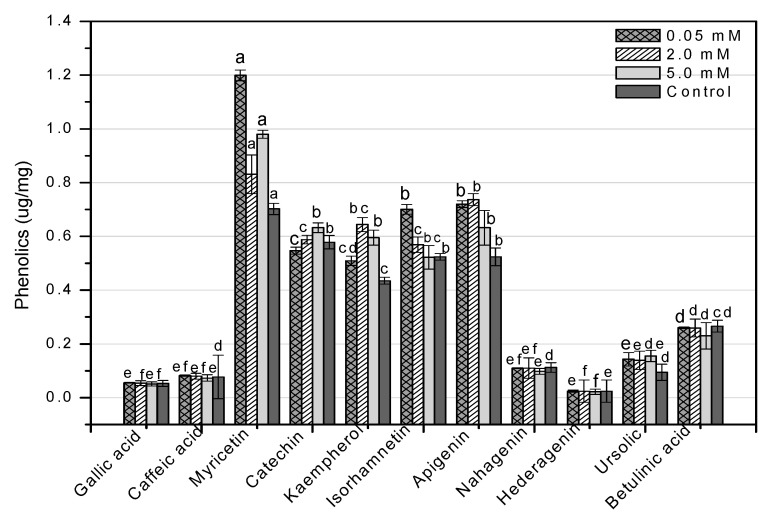
HPLC-based quantification of phenolic compounds in *F. indica* calli elicited with seven different concentrations of aluminum chloride along with control. Mean values with standard errors (± SE) and bars labeled with different letters (LSD values) exhibit significant variation (α < 0.05).

**Figure 14:**
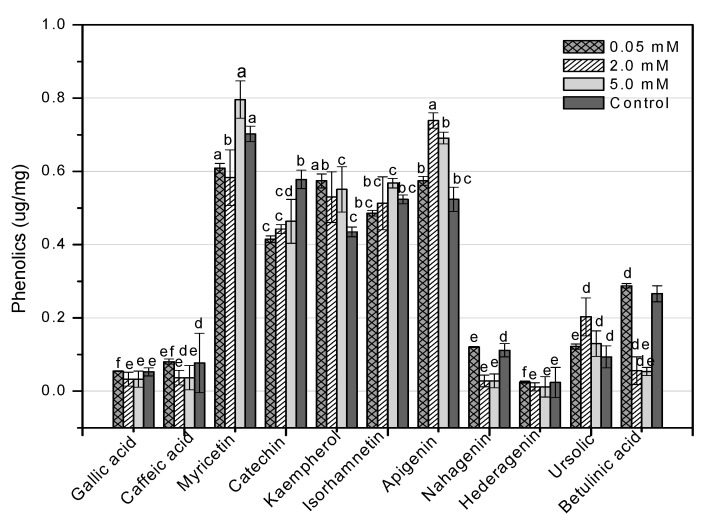
HPLC-based quantification of phenolic compounds in *F. indica* calli elicited with seven different concentrations of cadmium chloride along with control. Mean values with standard errors (± SE) and bars labeled with different letters (LSD values) exhibit significant variation (α < 0.05).

HPLC is an essential tool for evaluating secondary metabolites that provide a robust fingerprint analysis of plant therapeutic compounds [11]. This study used HPLC analysis to look into 11 different phenolic compounds in *F. indica* callus cultures provoked with AlCl_3_ and CdCl_2_ heavy metal salts (Table A1). In response to all AlCl_3_ concentrations, there was a significant increase in myricetin content. In reaction to 0.05 mM, the maximum enhancement (1.70-fold) was reported (Figure 13). Apigenin was enhanced similarly (1.37-fold, compared to control). Higher AlCl_3_ concentrations raised kaempferol levels (1.48-fold, compared to control). Isorhamnetin levels rose in response to reduced AlCl_3_ concentrations (1.33-fold, compared to control) (Figure 13). The content of kaempferol and apigenin was significantly increased at all CdCl_2_ doses. At 0.05, the maximum enhancement for kaempferol (1.31-fold compared to control) was reached, whereas, at 2 mM, the maximum enhancement for apigenin (1.41-fold compared to control) was achieved. The content of myricetin was marginally enhanced when the CdCl_2_ concentration was raised (1.13-fold, compared to control). Higher concentrations of CdCl_2_ increased isorhamnetin levels, although only to a lesser extent (1.08-fold, compared to control) (Figure 14). These four phenolics are all polyphenols, which are also known as flavonoids. The rest of the phenolics (simple phenols), also known as phenolic acids, showed no significant rise or decrease in response to the heavy metal amounts studied. These results show that AlCl_3_ and CdCl_2_ have enhancing effects on flavonoids. In diverse cancer cell lines, phenolic substances such as myricetin, apigenin, catechin, kaempferol, and isorhamnetin decrease oncogenes, reduce antioxidative stress, induce apoptosis, and stop the cell cycle [13].

## 3. Materials and Methods

### 3.1. Elicitation of Callus Culture with AlCl_3_ and CdCl_2_

Stems from one-month-old in vitro-germinated plantlets grown on solid MS (Murashige and Skoog 1962) medium (hormone-free) were selected as an explant source for callogenesis, as described by [13]. The explants were collected and aseptically cultured on an MS medium containing 3% sucrose and 0.8% agar, and augmented with 1.0 mg/L of thidiazuron (TDZ) at pH level 5.6 to induce callus formation. The medium was autoclaved at 121 °C for 20 min. The cultures were maintained at a 25 ± 2 °C temperature with a 70% relative humidity and a 16/8 h (light/dark) photoperiod providing an average illumination of 40 μmol/m^2^/s (Philips TLD 35 white light tubes). Fresh calli (0.5 g FW) were obtained and grown on MS media supplemented with AlCl_3_ and CdCl_2_ at 7 different doses (0.01, 0.05, 0.1, 0.5, 1.0, 2.0, and 5.0 mM) along with 1.0 mg/L of TDZ. The calli on the same MS medium fortified with TDZ (1.0 mg/L) only were used as the control group. The cultures were placed at 25 ± 2 °C in a growth room having a 16/8 h photoperiod, light intensity of 40 μmol/m^2^/s, and approximately 70% relative humidity. After 35 days of growth, the calli were harvested and gently kept on filter paper to detach media or normalize water content before fresh weight determination. After that, calli were oven-dried for dry weight determination and subsequently ground for further phytochemical analysis.

### 3.2. Determination of Total Phenolic Content (TPC) and Flavonoid Content (TFC)

The sample extraction for phytochemical analysis, i.e., total phenolic contents (TPCs) and total flavonoid contents (TFCs), was performed according to the protocol described by [61]. Dried samples (50 mg) were ground into powder, immersed in 500 µL of MeOH (Sigma Aldrich, Saint Quentin Fallavier, France), and sonicated for 60 min at 25 °C with a 45 kHz ultrasonic frequency (ElmaTM E plus 40H, Elma Schmidbauer GmbH, Singen, Germany). Vortexing for 5 min was used to collect extract, followed by centrifugation at 10,000 rpm for 15 min (Spectrafuge^TM^ 24D microcentrifuge, Labnet international, Edison, NJ, USA). The supernatant was filtered using a syringe and decanted into sterile storage tubes (1.5 mL Eppendorf tubes) at 4 °C.

The Folin–Ciocalteu reagent method (FCRM) was used for the assessment of total phenolic contents (TPCs), as per the method of [62]. For TPC determination, 90 μL of the Folin–Ciocalteu reagent (10× diluted in deionized distilled water) was added to each well of 96-well microplates containing 20 μL of the samples and allowed to react. This was followed by adding 90 μL of sodium carbonate (6 g/100 mL of distilled water) to each sample mixture, swirled gently, and finally allowed to stand for 90 min at room temperature. After incubation, the absorbance of the reaction mixture was measured spectrophotometrically at 630 nm using a microplate reader (ELx800BioTek, BioTek Instruments, Colmar, France). To plot the calibration curve (R^2^ = 0.967), gallic acid (0–50 μg/mL) was employed as standard. TPC was expressed as gallic acid equivalent (mg GAE/g) of DW.

Total phenolic production was calculated by using the following formula and expressed in mg/L.

Total phenolic production (mg∕L) = DW (g∕L) × TPC (mg∕g)(1)

Total flavonoids content was determined according to the aluminum chloride colorimetric method described by [63]. Briefly, 10 μL of aluminum trichloride solution (10 g/L of distilled water) and 10 μL (1 M) of potassium acetate (98.15 g/L of distilled water) were added to the reaction wells of a 96-well plate, containing 20 μL of the samples. The final reaction volume was raised to 200 μL by adding 160 μL of distilled water and incubated for 30 min at room temperature. The solution was mixed well, and finally, the change in absorbance was recorded at 415 nm with a microplate reader (ELx800BioTek, BioTek Instruments, Colmar, France). To plot the graph, quercetin (0–50 µg/mL) was used for standardized calibration (R^2^ = 0.967). TFC was taken as quercetin equivalents (mg QE)/g for expression of DW.

Total flavonoid production was calculated by using the following formula and expressed in mg/L.

Total flavonoid production (mg∕L) = DW (g∕L) × TFC (mg∕g)(2)

### 3.3. Determination of SOD and POD Activities

Extraction from a fresh sample was performed using the protocol of [64]. Briefly, fresh callus samples (0.1 g) were grounded in a mortar and pestle with 1 mL of extracting K-buffer (50 mM, pH 7) containing 1% polyvinylpyrrolidone (PVP). Acquired extracts were homogenized and subsequently centrifuged at 14,000 rpm for 30 min to separate the supernatant from cell debris. The supernatant was carefully removed with a micropipette and transferred into a new Eppendorf tube, and the remaining pellet was discarded. The supernatant fraction collected after centrifugation was then analyzed for analysis of POD and SOD.

Peroxidase (OD) assay was assessed by using the protocol of [65] with slight modifications. The reaction mixture of 200 μL was prepared by mixing 40 μL (50 mM) of K-phosphate buffer (pH 7), 20 μL of (100 mM) guaiacol (10×), 100 μL of distilled water, and 20 μL (27.5 mM) of H_2_O_2_ (10×), along with 20 μL of enzyme extract. An equal amount of all reagents was used as a control, excluding sample extract. After that, absorbance activity was determined spectrophotometrically at 470 nm with a 20 s gap using a microplate reader (ELx800BioTek, BioTek Instruments, Colmar, France). The enzymatic activity was measured using the formula given below:

A = ELC,(3)

where A = absorbance, E = extinction coefficient (6.39 mM^−1^ cm^−1^), L = length of each wall (0.25 cm), C = concentration of enzyme (value of C measured in mM/min/mg-FW), and FW = fresh weight of the sample.

Superoxide dismutase activity (SOD) was carried out using Giannoplolitis and Ries’ protocol [66]. The reaction mixture of 200 μL was prepared in a 96-well microplate containing all the required reagents that include 78 μL (50 mM) of phosphate buffer of pH 7, 20 μL (1 mM) of EDTA, 20 μL (130 mM) of methionine, 20 μL (0.75 mM) of NBT, and 2 μL (0.02 mM) of riboflavin, along with 60 μL of enzyme extract. Similarly, a blank was also prepared by mixing these chemicals, excluding fresh sample extract. This reaction mixture was exposed to fluorescent light for 7 min followed by OD measurement at 660 nm using a microplate reader (ELx800BioTek, BioTek Instruments, Colmar, France). The Equation (3) was opted for measuring enzymatic activity.

### 3.4. Determination of Free Radical Scavenging Assay (DPPH)

Free radical scavenging activity (FRSA) was measured using 2,2-diphenyl-1-picrylhydrazyl (DPPH) for the determination of antioxidant potential, as described by [67]. The stock reagent solution was prepared by dissolving 3.2 mg of DPPH in 100 mL of methanol and stored in a refrigerator until use. Briefly, 180 μL of 2,2-diphenyl-1-picrylhydrazyl (DPPH) reagent was added to the entire row of wells containing 20 μL of the samples to obtain the final concentrations of 200 μL. The OD was recorded at 517 nm using a microplate reader (ELx800BioTek, BioTek Instruments, Colmar, France) after 1 h of incubation in the dark at room temperature. The antioxidant potential of each biological sample was calculated as % DPPH discoloration, calculated by the following formula:

% scavenging = (Abc − Abs/Abc) × 100(4)

where Abc = absorbance of the control and Abs = absorbance of the sample

### 3.5. HPLC Quantification

**Figure 15:**
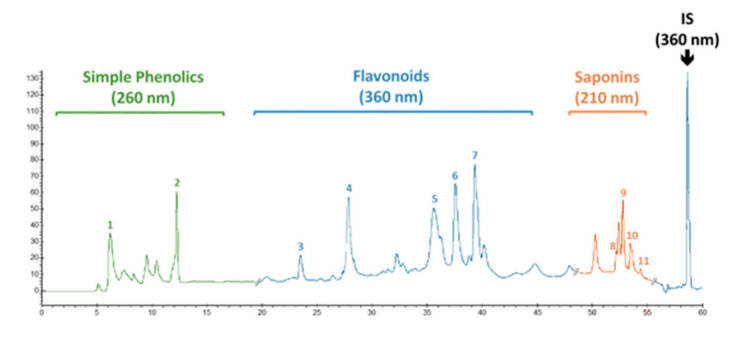
Typical HPLC chromatogram showing the presence of simple phenolics (gallic acid (1) and caffeic acid (2) recorded at 260 nm), flavonoids (catechin (3), myricetin (4), kaempferol (5), isorhamnetin (6), and apigenin (7) recorded at 360 nm), and saponins (hederagenin (8), betulinic acid (9), ursolic acid (10), and nahagenin (11) recorded at 210 nm) in in vitro (callus) culture of *F. indica.* 5-methoxyflavone (0.2 µg/mL) was used as an internal standard (detection set at 360 nm).

High-performance liquid chromatography (HPLC) was employed to quantify the presence of pharmaceutically important phenolic and flavonoid compounds in calli cultures of *F. indica.* The powdered calli samples, harvested at week 5, were analyzed through HPLC. An extract of dried material was prepared in 80% *v/v* (20 mL) methanol (aqueous). Then, extraction was carried out in an ultrasonic bath, USC 1200^TH^ (Prolabo Prolabo, Fontenay-sous-Bois, France) with inner dimension: 300 mm × 240 mm × 200 mm, equipped with an electrical power of 400 W (i.e., acoustic power of 1 W/ cm^2^), a maximal heating power of 400 W and variable frequencies, equipped with a digital timer, and a frequency and a temperature controller having a 30 kHz frequency for 1 h at 25 ± 2 °C. Following extraction, centrifugation of the samples was performed, and the supernatant was filtered with a 0.45 μm syringe filter (Millipore) before HPLC analysis. Phytochemical analysis was carried out using a Varian liquid chromatographic system (Varian, Les Ulis, France) composed of a Varian Prostar 230 pump, Metachem Degasit, Varian Prostar 410 autosampler, and Varian Prostar 335 Photodiode Array Detector (PAD), and it was controlled by Galaxie version 1.9.3.2 software. The reference standards used were gallic acid, caffeic acid, myricetin, catechin, kaempferol, isorhamnetin, apigenin, nahagenin, hederagenin, ursolic acid, and betulinic acid, purchased from Sigma Company, USA. A Purospher (Merck Chemical, Saint-Quentin Fallavier, France) RP-18 column (250 mm × 4.0 mm. id; 5 μm) was utilized for separation, and separation was performed at 35 °C. The mobile phase consisted of two solvents, solvent A (0.2% acetic acid in water) and solvent B (methanol). For mobile phase variation, a nonlinear gradient was applied with a flow rate of 0.8 mL/min as follows: from 0 to 40 min of A–B: 90:10 (*v/v*) to 30:70 (*v/v*), from 41 to 50 min of A–B: 30:70 (*v/v*) to 0:100 (*v/v*), and A–B: 0:100 (*v/v*) from 51 to 60 min. A UV-Vis spectrophotometer performed detection at 260 nm for simple phenolics, 360 nm for flavonoids, and 210 nm for saponins (Figure 15). The phenolic compounds were identified based on their comparison with the retention time and UV spectra to reliable reference standards. Quantification was performed using 5-points calibration curves of each standard with a correlation coefficient of at least 0.998. The quantifications were recorded using calibration curves and retention times of corresponding reference standards. All the samples were assayed in triplicate, and the results were expressed as micrograms per milligram of DW of the sample.

### 3.6. Experimental Design and Data Analysis

To investigate the effect of AlCl_3_ and CdCl_2_ elicitors on callus culture, seven concentrations of each elicitor and two controls as treatments were adopted under a randomized complete block design. All experimental results were means of three independent replicates. One-way ANOVA was used to test statistical differences, followed by Tukey’s HSD for post hoc analysis (Minitab statistical package 17, State College, PA, USA). Differences were considered significant at *p* < 0.05. Data were also evaluated using Pearson’s correlation coefficients to identify relationships between phenolic contents and selected antioxidant activities of *F. indica* calli. All the figures were made using the Origin Pro 2017 package (OriginLab, Northampton, MA, USA). All the data were represented as mean with standard error.

## 4. Conclusions

This study aimed to develop an effective elicitation technique for inducing biomass and metabolite biosynthesis in *F. indica* callus cultures. Heavy metal salts AlCl_3_ and CdCl_2_ were discovered to have considerable effects on biomass and phytochemicals, as well as antioxidative enzyme activity. Overall, AlCl_3_ produced the maximum amount of fresh weight biomass, phenolics, and flavonoids. HPLC examination revealed that AlCl_3_-mediated cultures accumulated the most chemicals compared to CdCl_2_ and control cultures. Similarly, AlCl_3_ was found to produce more free radical scavenging activities and antioxidant enzyme activities (SOD and POD) than cadmium chloride. Higher quantities of both elicitors, on the other hand, were found to have inhibitory effects on practically all of the parameters studied. As a result of this study’s practical approach, instead of direct extraction from the wild, key phytochemicals can be produced, reducing the risk of extinction for this species. To remove hazardous metals, chemical precipitation or filtering is currently employed in industry. As a result, we anticipate that if industrial use is desired, these techniques will be able to remove residues of metals in the extracts. However, further high-throughput investigations are needed to decode the molecular mechanisms that increase metabolite synthesis when heavy metals are elicited.

## Data Availability

All the data are included in the present study.

## Appendix A

**Table A1:** HPLC-based quantification of pharmacologically important phenolic compounds in *F. indica*.

Compounds Quantified (µg/mg-DW)	0.01 mM		0.05 mM		0.1 mM		0.5 mM		1.0 mM		2.0 mM		5.0 mM		0mM
	AlCl_3_	CdCl_2_	AlCl_3_	CdCl_2_	AlCl_3_	CdCl_2_	AlCl_3_	CdCl_2_	AlCl_3_	CdCl_2_	AlCl_3_	CdCl_2_	AlCl_3_	CdCl_2_	Control
GALLIC ACID	0.062 ± 0.022	0.061 ± 0.005	0.055 ± 0.001	0.055 ± 0.001	0.056 ± 003	0.062 ± 0.001	0.056 ± 0.017	0.044 ± 0.0012	0.057 ± 0.023	0.035 ± 0.001	0.054 ± 0.009	0.033 ± 0.019	0.051 ± 0.008	0.033 ± 0.022	0.053 ± 0.011
CAFFEIC ACID	0.097 ± 0.012	0.094 ± 0.008	0.082 ± 0.002	0.081 ± 0.007	0.084 ± 0.010	0.095 ± 0.001	0.083 ± 0.0057	0.059 ± 0.0064	0.086 ± 0.028	0.040 ± 0.032	0.080 ± 0.011	0.037 ± 0.020	0.073 ± 0.012	0.037 ± 0.033	0.077 ± 0.081
MYRICETIN	0.655 ± 0.014	0.799 ± 0.023	1.199 ± 0.020	0.609 ± 0.013	0.827 ± 0.019	0.771 ± 0.002	1.129 ± 0.017	0.777 ± 0.0058	0.662 ± 0.034	0.725 ± 0.009	0.831 ± 0.072	0.583 ± 0.076	0.980 ± 0.015	0.796 ± 0.051	0.702 ± 0.021
CATECHIN	0.522 ± 0.09	0.476 ± 0.013	0.547 ± 0.013	0.415 ± 0.009	0.623 ± 0.023	0.444 ± 0.0023	0.533 ± 0.021	0.566 ± 0.0031	0.589 ± 0.017	0.560 ± 0.051	0.588 ± 0.016	0.443 ± 0.012	0.632 ± 0.018	0.464 ± 0.060	0.578 ± 0.025
KAEMPHEROL	0.560 ± 0.016	0.476 ± 0.019	0.509 ± 0.017	0.574 ± 0.019	0.456 ± 0.021	0.481 ± 0.0038	0.605 ± 0.0057	0.528 ± 0.0018	0.626 ± 0.011	0.523 ± 0.068	0.644 ± 0.026	0.530 ± 0.069	0.595 ± 0.028	0.551 ± 0.062	0.435 ± 0.013
ISORHAMNETIN	0.674 ± 0.08	0.422 ± 0.011	0.701 ± 0.018	0.486 ± 0.008	0.567 ± 0.031	0.408 ± 0.0013	0.641 ± 0.0036	0.662 ± 0.0017	0.499 ± 0.026	0.670 ± 0.015	0.569 ± 0.029	0.513 ± 0.072	0.522 ± 0.044	0.568 ± 0.013	0.524 ± 0.012
APIGENIN	0.883 ± 0.010	0.601 ± 0.015	0.720 ± 0.013	0.574 ± 0.012	0.660 ± 0.002	0.626 ± 0.0028	0.804 ± 0.011	0.681 ± 0.0015	0.626 ± 0.034	0.670 ± 0.018	0.737 ± 0.022	0.739 ± 0.021	0.632 ± 0.065	0.691 ± 0.016	0.524 ± 0.033
NAHAGENIN	0.142 ± 0.020	0.146 ± 0.029	0.110 ± 0.001	0.121 ± 0.001	0.118 ± 0.005	0.145 ± 0.0023	0.120 ± 0.024	0.065 ± 0.0019	0.127 ± 0.051	0.031 ± 0.027	0.110 ± 0.038	0.028 ± 0.016	0.098 ± 0.011	0.028 ± 0.019	0.112 ± 0.018
HEDERAGENIN	0.028 ± 0.017	0.028 ± 0.002	0.024 ± 0.004	0.025 ± 0.003	0.025 ± 0.009	0.028 ± 0.0015	0.025 ± 0.023	0.018 ± 0.0040	0.026 ± 0.011	0.013 ± 0.025	0.024 ± 0.041	0.012 ± 0.011	0.022 ± 0.010	0.012 ± 0.028	0.024 ± 0.041
URSOLIC ACID	0.201 ± 0.001	0.114 ± 0.010	0.143 ± 0.025	0.122 ± 0.007	0.148 ± 0.011	0.135 ± 0.0017	0.134 ± 0.031	0.173 ± 0.0034	0.087 ± 0.026	0.195 ± 0.022	0.139 ± 0.034	0.203 ± 0.052	0.155 ± 0.021	0.130 ± 0.035	0.094 ± 0.030
BETULINIC ACID	0.338 ± 0.005	0.349 ± 0.003	0.260 ± 0.003	0.288 ± 0.006	0.280 ± 0.009	0.348 ± 0.0011	0.286 ± 0.0057	0.146 ± 0.0011	0.304 ± 0.0173	0.061 ± 0.020	0.259 ± 0.033	0.056 ± 0.038	0.230 ± 0.049	0.054 ± 0.011	0.266 ± 0.022
